# Brain Regions Involved in Underlying Syntactic Processing of Mandarin Chinese Intransitive Verbs: An *f*MRI Study

**DOI:** 10.3390/brainsci11080983

**Published:** 2021-07-24

**Authors:** Xin Wang, Shiwen Feng, Tongquan Zhou, Renyu Wang, Guowei Wu, Fengshan Ni, Yiming Yang

**Affiliations:** 1 School of Humanities and Arts, China University of Mining and Technology, Xuzhou 221116, China; super_wangxin2010@163.com; 2 School of Linguistic Sciences and Arts, Jiangsu Normal University, Xuzhou 221009, China; jsnuwangry@163.com (R.W.); 2020170388@jsnu.edu.cn (G.W.); 3 School of Liberal Arts, Nantong University, Nantong 226019, China; 4 Collaborative Innovation Center for Language Ability, Jiangsu Normal University, Xuzhou 221009, China; zhoutongquan@126.com; 5 School of Translation Studies, Qufu Normal University, Qufu 276800, China; 6 School of Chinese Language and Literature, Hubei University, Wuhan 430062, China; 201801110200011@stu.hubu.edu.cn

**Keywords:** unaccusative verb, unergative verb, syntactic properties, Mandarin Chinese, *f*MRI

## Abstract

According to the Unaccusative Hypothesis, intransitive verbs are divided into unaccusative and unergative ones based on the distinction of their syntactic properties, which has been proved by previous theoretical and empirical evidence. However, debate has been raised regarding whether intransitive verbs in Mandarin Chinese can be split into unaccusative and unergative ones syntactically. To analyze this theoretical controversy, the present study employed functional magnetic resonance imaging to compare the neural processing of deep unaccusative, unergative sentences, and passive sentences (derived structures undergoing a syntactic movement) in Mandarin Chinese. The results revealed no significant difference in the neural processing of deep unaccusative and unergative sentences, and the comparisons between passive sentences and the other sentence types revealed activation in the left superior temporal gyrus (LSTG) and the left middle frontal gyrus (LMFG). These findings indicate that the syntactic processing of unaccusative and unergative verbs in Mandarin Chinese is highly similar but different from that of passive verbs, which suggests that deep unaccusative and unergative sentences in Mandarin Chinese are both base-generated structures and that there is no syntactic distinction between unaccusative and unergative verbs in Mandarin Chinese.

## 1. Introduction

**Figure 1:**
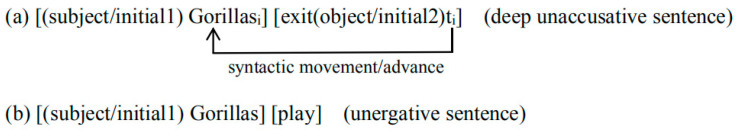
The schematic diagrams of the generating process of deep unaccusative sentences and unergative sentences. See (a) for the example of a deep unaccusative sentence and (b) for the example of an unergative sentence.

**Figure 2:**
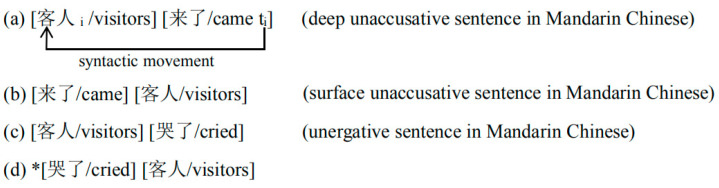
As shown in Figure 2, in Mandarin Chinese, the argument of unaccusative verbs can appear in the subject or object position, but the argument of the unergative verbs can only appear at the subject position. When in the subject position, the argument forms a deep unaccusative sentence, and when in the object position, it forms a surface unaccusative sentence. See (a) for the example of a deep unaccusative sentence in Mandarin Chinese, (b) for the example of a surface unaccusative sentences in Mandarin Chinese, (c) for the example of an unergative sentence in Mandarin Chinese. (d) The asterisk means that the sentence after it doesn’t hold.

Verbs are generally considered to be the core of a sentence as they determine the syntactic structure of the sentence where they appear. For instance, a sentence containing the verb *cry* has no object, whereas a sentence containing the verb *hit* is determined to have one. As such, verbs are conventionally divided into transitive and intransitive ones [1,2]. The division of verb subcategories is not only an important issue in theoretical linguistic research but also a focal topic in neurolinguistics and psycholinguistics [3,4]. One of the most influential theories associated with the subcategorization of verbs is the Unaccusative Hypothesis proposed by Perlmutter [5], which posits the view that intransitive verbs can be divided into unaccusative and unergative ones, concerning the distinct base-generated positions of their subjects. In the surface structure, deep unaccusative sentences and unergative sentences have the same word order, where the noun phrase is followed by the verb. However, as shown by (a) in Figure 1, *gorillas*, the surface subject of the unaccusative verb *exist*, is originally in the initial2 position (an object position) and then moves to the initial1 position (a subject position). By comparison, (b) in Figure 2 shows that as the surface subject of the unergative verb *play*, *gorillas* is base-generated in the initial1 position. Therefore, in the syntactic generation process, the subject of the unaccusative verb involves advance (referred to as movement in subsequent research) from the initial 2 position to the initial1 position, while the subject of the unergative one does not involve this syntactic operation. Based on the Unaccusative Hypothesis, many researchers argue that intransitive verbs can be divided into unaccusative and unergative categories, and have accounted for this generalization by proposing different syntactic generation process for each type of intransitive verb. Unaccusative verbs have an internal argument which is base-generated in the object position and then moves to the subject position. Whereas, unergative verbs have an external argument that is base-generated in the subject position [6,7,8,9]. Therefore, even though the surface structures of deep unaccusative sentences and unergative sentences are both NP-V, the former are derived structures undergoing a syntactic movement and the latter are base-generated structures.

The Unaccusative Hypothesis has been examined by numerous empirical studies revealing that the syntactic processes underlying unaccusative verbs and unergative verbs are different. In experiments on children’s first language acquisition, using the picture–sentence matching task, it has been documented that children can generate unaccusative sentences using an NP-V structure or a V-NP structure at earlier times, but can only use an NP-V structure to generate unergative sentences [10,11,12]. This is indicative that the argument in the surface subject position of the deep unaccusative sentence is originally in the internal argument (object) position, but that of the unergative one is initially in the external argument (subject) position.

Further, second language (L2) acquisition studies indicate that it is more difficult for L2 learners to comprehend an unaccusative structure than an unergative one. This suggests that processing an unaccusative sentence is more complicated than processing an unergative sentence, presumably because the former must undergo an additional syntactic movement during syntactic processing [13,14].

Moreover, in aphasic studies using lexical naming tasks [15,16] and picture–sentence matching tasks [17,18], patients, relative to controls, produce fewer unaccusative verbs and sentences, with a higher error rate and longer response time. These results lend well to the hypothesis that brain damage associated with aphasia is related to syntactic movement, which would induce difficulty in processing unaccusative verbs and sentences.

In addition, functional magnetic resonance imaging (*f*MRI) studies have revealed greater activation in the left inferior frontal gyrus and the left posterior temporal gyrus, elicited by the processing of unaccusative verbs in comparison to unergative verbs [19,20,21,22]. These results suggest the possibility of separable neural mechanisms invoked during unaccusative and unergative verb processing, which have supported the division of unaccusative verbs and unergative verbs at the syntactic level.

Although there is much theoretical and experimental evidence showing that unaccusative verbs and unergative verbs have different syntactic properties, some debates remain concerning unaccusative and unergative verbs in Mandarin Chinese, a non-inflectional language, and whether they belong to different subcategories syntactically. On the one hand, supporters argue that unaccusative verbs in Mandarin Chinese can appear in both NP-V structures and V-NP structures, which respectively form deep unaccusative sentences (see (a) in Figure 2), and surface unaccusative sentences (see (b) in Figure 2). However, unergative verbs in Mandarin Chinese can only appear in NP-V structures (see (c) and (d) in Figure 2). This difference indicates that the subject of Mandarin deep unaccusative sentences is base-generated in the object position and experiences an extra syntactic movement, while that of Mandarin unergative sentences does not [23,24,25]. On the other hand, however, opponents point out that Mandarin surface unaccusative sentences violate the rule that unaccusative verbs cannot assign the objective case to the deep object. It is thus no wonder that there emerges the view that Mandarin Chinese is a language without case marking and that the deep unaccusative and unergative sentences are both base-generated structures that do not experience a syntactic movement. This suggests that there is no syntactic distinction between Mandarin unaccusative verbs and unergative verbs and that the Unaccusative Hypothesis may not hold well in the case of Mandarin Chinese [26].

A possible way to resolve the theoretical divergence here is to directly compare the neural processing of deep unaccusative sentences with unergative sentences and find out whether the syntactic movement involved in deep unaccusative sentences has a unique neural mechanism. Therefore, similar to previous studies [19,20,21,22], the present study adopts the *f*MRI technique to investigate how Mandarin deep unaccusative sentences and unergative sentences are processed. By comparing the processing neural mechanisms of these two types of sentences, it is also possible to explore whether the Unaccusative Hypothesis, an important linguistic theory related to intransitive verbs, holds for all languages or not.

**Figure 3:**
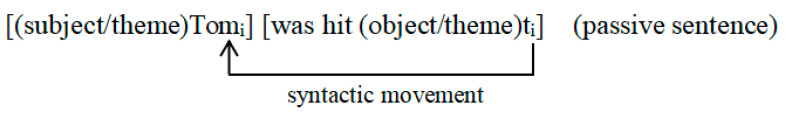
The schematic diagrams of the generating process of a passive sentence.

Theoretical linguists have claimed that passive verbs and unaccusative verbs are similar subcategories of intransitive ones, suggesting the syntactic generation process of deep unaccusative sentences is similar to that of passive ones [24,27,28]. Based on the analysis above, the passive verb’s argument is assigned to a patient theta role and is base-generated in the object position. Consequently, in the syntactic generation process, the passive verb’s internal argument must move to the surface subject position of the sentence (see Figure 3), as does the accusative verb’s internal argument. This theoretical assumption has received some empirical support. For example, Kim [29] explored the priming effect in passive and active sentences induced by deep unaccusative sentences, using the syntactic priming paradigm, and reported a significant priming effect for passive sentences compared to active sentences, suggesting that deep unaccusative and passive sentences quite possibly share a similar syntactic process. Additionally, syntactic movement in passive sentence processing has been demonstrated by several *f*MRI studies, which found that the neural processing of passive sentences induced activation in the left inferior frontal gyrus (LIFG) and the left posterior temporal gyrus compared to that of active sentences. These brain regions play a key role in syntactic movement, which is involved in passive sentence processing rather than that of the active one [30,31,32]. Therefore, to investigate whether the subject of deep unaccusative sentences experiences an additional syntactic movement compared with that of unergative sentences in Mandarin Chinese, it is important to explore the neural processing of passive sentences and compare it with Mandarin deep unaccusative and unergative sentences.

## 2. Materials and Methods

### 2.1. Participants

Sixteen participants (undergraduate or masters students, 8 females, age range: 20–26) were recruited from a university in China to participate in this study. All of them had a normal or adjusted-to-normal vision and no neurological or psychiatric medical history. All the participants were right-handed, as assessed with the Edinburgh Handedness Inventory [33]. They were all native Mandarin Chinese speakers and EFL learners. Before the experiment, all participants completed a practice session and signed informed consent forms issued by the Ethical Committee of the Institute of Linguistics at Jiangsu Normal University. The participants received payment for their participation.

### 2.2. Materials and Design

The experimental materials included three categories of Chinese verbs: 8 unaccusative verbs, 8 unergative verbs, and 8 transitive verbs. The transitive verbs were used to form the corresponding passive verbs with a functional word *被* (bèi). The unaccusative and unergative verbs were selected in two stages. First, the scope for selection was confined by collecting examples of these two kinds of verbs appearing in previous studies of Chinese (e.g., [23,24,25,34]). Next, verbs were chosen using the criterion that unaccusative verbs can appear in both NP-V and V-NP structures without a surface subject, while unergative ones can only appear in NP-V structures. To match the animacy of the arguments, unaccusative verbs (e.g., *碎* (suì)/break, *裂* (liè)/split) with inanimate internal arguments were excluded. In addition, to eliminate the influence imposed by the optional thematic frames, the alternating unaccusative verbs (e.g., *繁荣* (fán róng)/boom) that could be used as transitive verbs were also excluded. Ultimately, 24 verbs were selected for the experiment, including 8 verbs for each category. The eight unaccusative verbs were *死* (sǐ)/ to die, *来* (lái)/ to come, *到* (dào)/to arrive, *去* (qù)/ to go, *跑* (pǎo)/depart, *倒* (dǎo)/ to fall, *逃* (táo)/to get away, and *走* (zǒu)/ to be away. The eight unergative verbs were *哭* (kū)/ to cry, *睡* (shuì)/ to sleep, *输* (shū)/ to lose, *赢* (yíng)/ to triumph, *醒* (xǐng)/ to wake up, *病* (bìng)/to go sickness, *渴* (kě)/ to go thirsty, *笑* (xiào)/ to laugh). Meanwhile, the eight transitive verbs selected to form passive sentences were *打* (dǎ)/ to hit, *救* (jiù)/ to save, *骂* (mà)/ to condemn, *抓* (zhuā)/ to grab, *杀* (shā)/ to kill, *吃* (chī)/ to eat, *夸* (kuā)/ to praise, and *骗* (piàn)/ to deceive.

Since log10 Word frequencies-Contextual Diversity (logW-CD, [35]) is a useful tool to match Chinese word frequency, it was adopted to frequency-match the three types of verbs in the experiment. An analysis of variance (ANOVA) revealed that the mean logW-CD of unaccusative verbs was 3.38 (SD = 0.46), that of unergative verbs was 3.23 (SD = 0.36), and that of transitive verbs was 3.30 (SD = 0.52), indicating no significant difference in the frequency of these verbs (F_2,21_ = 2.83, *p* = 0.82). Sample sentences for the different conditions are listed in Table 1.

Every verb appeared in six different sentences, four of which were correct, and two of which were incorrect. Considering that the experimental task was sentence comprehension by the visual presentation, the characters of each sentence were controlled, that is, each sentence was constructed of five characters. Moreover, the syntactic structure of each sentence was parallel. The syntactic structures of the Mandarin deep unaccusative sentences were formed as double-character noun + single-character adverb *全* (quán)/all + single-character unaccusative verb + aspect particle *了* (le); the syntactic structures of Mandarin unergative sentences were formed as double-character noun + single-character adverb *全*/all + single-character unergative verb + aspect particle *了* (le); the syntactic structures of Mandarin passive sentences were formed as: double-character noun + single-character preposition *被* + single-character transitive verb + aspect particle *了* (le). As a result, the syntactic structures of the three types of sentences were all subject + predicate. For each category of sentences, the subjects of the correct ones were animate nouns, and the subjects of incorrect ones were inanimate nouns, which could collocate with the corresponding verb syntactically but could not do so semantically. Additionally, to maximally prevent the participants from judging the presented sentence based solely on the animacy of the initial subject, the present experiment included filler sentences, the syntactic structure of which was formed by double-character noun + single-character preposition *全*/all + single-character adjective + aspect particle *了* (le). For the correct filler sentences, subjects included animate and inanimate nouns.

A standard survey of sentence acceptability was conducted before the *f*MRI experiment. Fifty native Chinese speakers participated in a pilot task to screen the stimuli. These participants were asked to rate the acceptability of sentences with a five-point Likert scale (1 represented very unacceptable, and 5 indicated very acceptable). Only the sentences scoring above 75% acceptability (i.e., the acceptability rating of the sentence was over 3.75) were selected as correct stimuli for the *f*MRI experiment, and only the sentences below 25% acceptability (i.e., the acceptability rating of the sentence was under 1.25) were selected as incorrect stimuli in the *f*MRI experiment. The acceptability ANOVA results of correct sentences of three types of sentences were deep unaccusative sentences M = 4.35 ± 0.19SD, unergative sentences M = 4.34 ± 0.14SD, and passive sentences M = 4.35 ± 0.12SD, signifying that there was no significant difference among correct sentence categories (F_2_, 147 = 0.1, *p* = 0.91). Additionally, we found no significant difference in an ANOVA of incorrect sentences (F_2_,147 = 0.4, *p* = 0.67); for deep unaccusative sentences, M = 1.13 ± 0.08SD; unergative sentences, M = 1.12 ± 0.09SD; and passive sentences, M = 1.14 ± 0.09SD.

**Figure 4:**
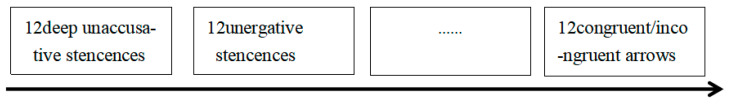
Experiment paradigm for *f*MRI scanning session.

A block design was used for *f*MRI data acquisition. In the *f*MRI scanning session, sentences (including deep unaccusative sentences, unergative sentences, passive sentences, and filler sentences) were divided into 4 blocks, each of which contained 12 trials (8 correct and 4 wrong sentences). Each trial lasted 1500 ms with a fixed intertrial interval (ITI) of 1500 ms where subjects viewed a fixation cross. Each block lasted 36 s (see Figure 4 for the *f*MRI scanning session). During the experiment, participants performed a comprehension task by pressing the right button if the sentence was correct or pressing the left one if the sentence was incorrect, as quickly as possible. To ensure participants completely processed the sentences presented to them, we told them in advance that they must read every sentence carefully before pressing the buttons in the formal experiment. The blocks were counterbalanced according to a Latin Square design. To detect how language was processed excluding nonverbal factors, the flanker task developed by Eriksen and Eriksen [36] was utilized to be a control task, where participants needed to press the left or right button, just as when they performed a language comprehension task. In this task, participants were asked to determine whether the central arrow was pointing in the same (congruent) or opposite (incongruent) direction.

### 2.3. Data Collection

The functional neuroimaging data were collected by the GE MR750 3T MRI machine at the Collaborative Innovation Centre for Language Ability, Jiangsu Normal University. The functional images were scanned with an EPI (echo-planar image) imaging sequence to obtain 34 slices of horizontal functional images. The specific scanning parameters were as follows: TE (echo time) = 30 ms, TR (repetition time) = 2000 ms, slice thickness = 4 mm, interval = 0 mm, FOV (field of view) = 200 mm, and matrix size = 64 × 64. The three-dimensional structural images were scanned using an SPGR (spoiled gradient recalled echo) imaging sequence to obtain 176 sagittal images. The specific scanning parameters were as follows: TE = 3.02 ms, TR = 26 ms, slice thickness = 1 mm, gap = 0 mm, FOV = 256 mm, and matrix = 256 × 256.

### 2.4. Data Analysis

**Figure 5:**
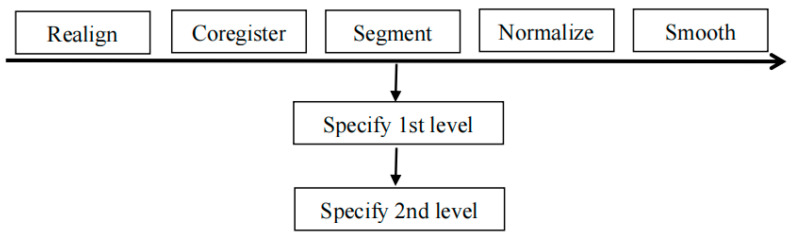
The progress flow of *f*MRI data analysis.

Analyses of variance (ANOVAs) were computed using SPSS (version 19.0) to compare differences in performance between the three-sentence conditions as assessed by accuracy and reaction time. For the reaction time, only the correct responses were counted. The *f*MRI data were analyzed with the SPM12 (Statistical Parametric Mapping 12) software package (http://www.fil.ion.ucl.ac.uk/spm/software/spm12/ (accessed on 1 July 2019) in MATLAB 2010 (MathWorks, Natick, MA, USA). After the first two dummy scans, which occurred in the first four seconds of the session, the remaining images were realigned to reduce the influence of head movement and the coregistration transformed the functional images. Spatial normalization was conducted for each participant; then, the segmentation divided the brain structure into three parts: gray matter, white matter, and cerebrospinal fluid. The functional images were spatially smoothed with an isotropic 8 mm FWHM (full width at half-maximum) Gaussian kernel. For individual statistical analysis (also named specify 1st level), a paired-samples *t*-test was performed to make a comparison between the neural mechanisms of passive sentences and deep unaccusative sentences, passive sentences and unergative sentences, and those of each sentence condition versus the control task. After this, group statistical analysis (also named specify 2nd level) superimposed the results of all subjects under each condition (see Figure 5 for the progress flow of *f*MRI data analysis).

A region of interest (ROI) analysis was conducted to explore the differences in the left superior temporal gyrus (LSTG) and the left middle frontal gyrus (LMFG) for the comparison between passive sentences and deep unaccusative sentences, passive sentences, and unergative sentences. The percentage signal changes of the LSTG and the LMFG activated by the different sentence conditions in the present experiment were contrasted using a paired samples *t*-test. An xjview (http://www.alivelearn.net/xjview (accessed on 1 July 2019) structural template image with MNI (Montreal Neurological Institute) coordinates was used for data reporting.

## 3. Results

### 3.1. Behavioral Results

According to the ANOVAs, the mean accuracies for deep unaccusative sentences, unergative sentences, and passive sentences were 94.66% (SD = 0.042), 94.01% (SD = 0.043), and 94.66% (SD = 0.036) respectively. As for deep unaccusative ones, the mean reaction time was 1012.54 ms (SD = 110.25). Additionally, the mean reaction time of unergative sentences was 1001.45 ms (SD = 109.60), and that of passive sentences was 1011.90 ms (SD = 108.57). The data showed no difference between conditions in accuracy (F_2_,45 = 0.14, *p* = 0.87) and in reaction time (F_2_,45 = 0.05, *p* = 0.95). The results thus demonstrate that there was no difference in task difficulty among the different sentence conditions. The paired samples *t*-test results show that for the mean accuracy and reaction time, there were significant differences between sentence condition and control task (for the mean accuracy, *t* (15) = −5.74, *p* < 0.001 and for the reaction time, *t* (15) = 13.28, *p* < 0.001), which indicates that processing the sentence condition was more complex than processing the control task (flanker).

### 3.2. fMRI Results

**Figure 6:**
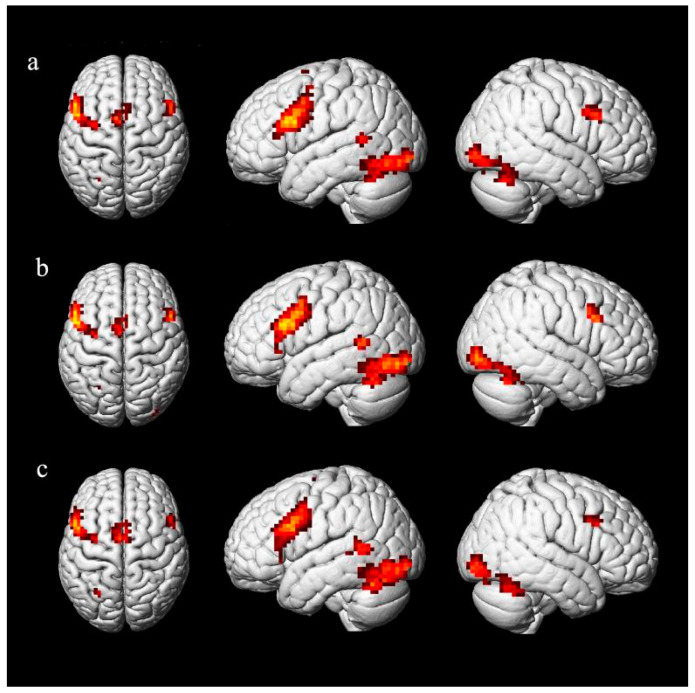
Regions of differential activation for unaccusative, unergative and passive sentences compared to control task (cluster FWE correction, *p* < 0.05). Notes: (**a**): deep unaccusative sentences > control task; (**b**): unergative sentences > control task; (**c**): passive sentences > control task.

The control task (flanker) was compared with each of the three sentence types. The comparison between deep unaccusative sentences and the control task (Unaccusative > Control) revealed activation in the bilateral occipital lobes, the bilateral fusiform gyrus, the LIFG, the LMFG, the left middle temporal gyrus (LMTG), and the right lingual gyrus (see Table 2 and Figure 6a). The comparison between unergative sentences and the control task (Unergative > Control) revealed activation in the bilateral occipital lobes, the left inferior temporal gyrus (LITG), the bilateral precentral gyrus, the bilateral inferior frontal gyrus, the LMFG, and the left inferior parietal lobe (see Table 2 and Figure 6b). The comparison between passive sentences and the control task (Passive > Control) revealed activation in the bilateral occipital lobes, the left precentral gyrus, the LIFG, the left inferior parietal lobe, and the left medial frontal gyrus (see Table 2 and Figure 6c).

**Figure 7:**
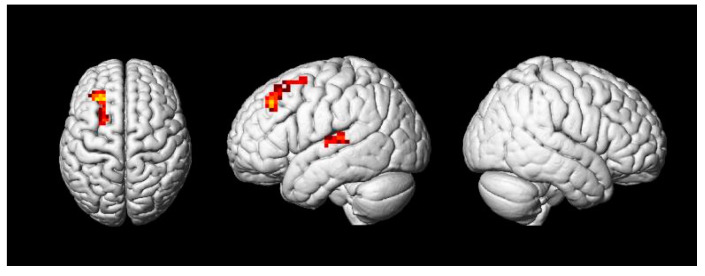
Regions of the null conjunction of the comparison between passive sentences and deep unaccusative sentences and the comparison between passive sentences and unergative sentences (cluster FWE correction, *p* < 0.05).

**Figure 8:**
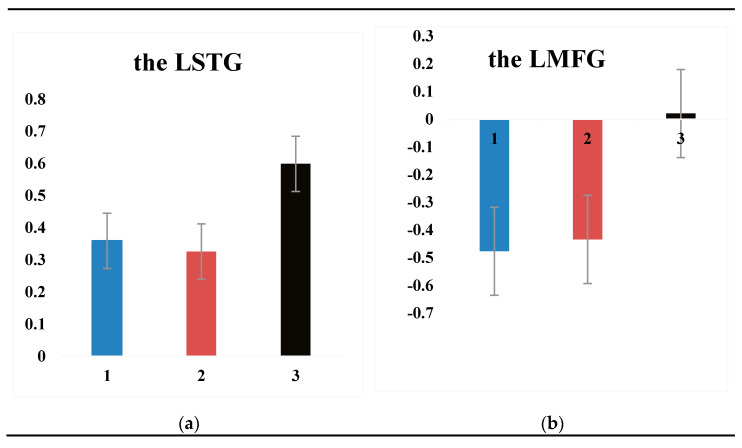
Percent signal changes extracted by ROI analysis on the left temporal lobes and the left middle frontal gyrus of deep unaccusative sentences, unergative sentences, and passive sentences. Notes: The peak location of the left superior temporal gyrus (LSTG, BA21/22) is at the point [7,33,37] (see **a**), and the peak location of the left middle frontal gyrus (LMFG, BA9) is at the point [31,34,38] (see **b**). The ROIs comprised of a 5 mm sphere. 1: deep unaccusative sentences; 2: Unergative sentences; 3: Passive sentences.

Furthermore, the result of the whole-brain analysis showed no significant difference in the neural processing of deep unaccusative sentences and unergative sentences, but passive sentences elicited greater activation comparing with them. Meanwhile, the aim of the present research was to detect whether or not the argument of deep unaccusative sentences was involved in an additional syntactic movement, just like that of passive sentences, compared to that of unergative sentences. Therefore, it was necessary to analyze the similarities or differences of argument processing between passive sentences and the other two kinds of sentences. To compare the activation related to the processing of passive sentences with that related to deep unaccusative sentences and unergative sentences processing, we performed a null conjunction analysis ([passive sentences > deep unaccusative sentences] ∩ [passive sentences > unergative sentences]), consulting past research [19,21,22]. The conjunction analysis showed activation in the LSTG and the LMFG (see Figure 7 and the conjunction condition in Table 3). For reference to studies carried out in the past [19,21,22], we conducted an ROI-based (region of interest-based) analysis to examine interactions between the three kinds of sentences in our research and two areas, which were the LSTG (peak location was at the point [7,33,37], BA21/22) and the LMFG (peak location was at the point [31,34,38], BA9). The ROIs comprised a 5 mm sphere. The specific results of the ROI analysis are shown in Figure 8. For the LSTG, significant differences were found between the passive sentences and the deep unaccusative sentences (*t* (15) = 2.70, *p* < 0.05) and the passive sentences and the unergative sentences (*t* (15) = 2.51, *p* < 0.05), but no significant difference was found between the deep unaccusative sentences and the unergative sentences (*t* (15) = 0.43, *p* = 0.67). For the LMFG, significant differences were also observed between passive sentences and deep unaccusative sentences (*t* (15) = 2.99, *p* < 0.01), and passive sentences and unergative sentences (*t* (15) = 2.68, *p* < 0.05). Nevertheless, no significant difference was found between deep unaccusative sentences and unergative sentences (*t* (15) = 0.29, *p* = 0.78).

## 4. Discussion

This *f*MRI study explored brain activity associated with the processing of unaccusative, unergative verbs, and passive verbs in sentence comprehension tasks in Mandarin Chinese. By exploring the processing neural mechanisms of the deep accusative, unergative and passive sentences, we carried out the revelation and comparison of syntactic processing among the three categories of sentences to detect whether or not the argument of deep unaccusative sentences was involved in an additional syntactic movement, just like that of passive sentences.

Our *f*MRI results showed that compared to the control task, the processing of deep unaccusative sentences, unergative sentences, and passive sentences in Mandarin Chinese elicited greater activation in the LIFG, the LSTG, and the LMTG. The results indicate that these regions might perform a significant function in processing language, which has consistently been supported by numerous studies exploring the neural mechanisms of language processing (e.g., [30,39,40,41,42]). In addition, the processing of the control condition elicited greater activation in the occipital lobe, which was related to visual processing [43]. Under the control condition, the participants needed to determine whether the central arrow was pointing in the same (congruent) or opposite (incongruent) direction, which might cost more visual processing. Therefore, the occipital lobe maybe reflected the more complex visual processing of the control condition. In contrast to the processing of Mandarin unergative sentences, that of Mandarin deep unaccusative sentences did not elicit more activation in any brain region. However, compared to these two types of sentences, Mandarin Chinese passive sentences that were proved to be linked to an additional syntactic movement elicited more activation in the LSTG and the LMFG. This further suggests that the processing of unaccusative and unergative verbs in Mandarin Chinese is highly similar and that the processing of the passive sentences is more complicated.

Relative to the other two kinds of verbs, Mandarin Chinese passive verb has different processing mechanisms in many aspects. Firstly, the subject of passive verb is a passive argument that is base-generated in the object position, which indicates that a syntactic movement operation and the reanalysis of the corresponding relationship between the passive theta role and the subject syntactic position may occur during the process of passive verb processing. Secondly, the form of passive verb is irregular as it is transformed from the corresponding transitive verb with a preposition, suggesting that the verb form processing of it is more complex. Thirdly, the passive verb often expresses an abstract semantic of suffering, indicating that the semantic processing of this kind of verb is also more complicated than those of unaccusative verb and unergative verb. Fourthly, the syntactic structure where Mandarin Chinese passive verb appears is optional as this type of verb can appear in both long and short passive sentences. Therefore, the processing of passive verb may touch upon syntactic structure selection.

### 4.1. The LSTG

In the present study, compared with Mandarin deep unaccusative and unergative sentences, Mandarin passive sentences activated the LSTG to a greater degree. Previous work has proposed that left temporal lobes are critical to language processing [38,44,45], and the left posterior temporal gyrus has also been found in many studies exploring the syntactic processing of passive sentences. For example, recent *f*MRI studies have found that passive sentences prompted more activation in the left posterior temporal gyrus compared with active sentences [30,32].

Additionally, previous studies have inferred three reasons for the left posterior temporal gyrus activation in sentence processing. The first reason attributes the region to syntactic movement. For example, in the language processing model of Friederici [46], the LSTG was linked to complex syntactic processing including movement. In light of the Uniformity of Theta Assignment Hypothesis (UTAH, [47]), the specific theta role appears in the corresponding base-generated position, i.e., the agent is base-generated in the external argument (subject) position while the base-generated position of the patient is in the internal argument (object) position. Meanwhile, the patient argument of passive sentences initially in the object position is mapped to the subject position. Consequently, in Mandarin Chinese, the subject of passive verbs should experience an additional syntactic movement from the object position to the subject position in the generation process. Theoretical accounts [24,28,48] and experimental studies [30,32] also agree that Mandarin Chinese passive sentences are structures derived by a syntactic movement, which is consistent with that of many other languages. According to previous theoretical and experimental research, the LSTG concerning passive sentence processing in the present experiment was likely related to the syntactic movement of the subject in passive sentences.

Of particular note is that the results indicated that contrasted with Mandarin deep unaccusative sentences and unergative sentences, Mandarin passive sentences elicited more activation in the LSTG, but no significant difference was found contrasting deep unaccusative sentences with unergative ones. These results showed that for Mandarin Chinese, the neural processing of deep unaccusative sentences and unergative sentences was highly similar but different from that of the passive ones, which then suggests that compared with the Mandarin passive sentences, the derived structures that underwent syntactic movement from the internal position to the external position, the Mandarin deep unaccusative sentences and unergative sentences were both base-generated structures, which further suggests that the arguments of Mandarin unaccusative and unergative verbs were both base-generated in the surface subject (or the external argument) position. Therefore, the analysis here indicates that intransitive verbs in Mandarin Chinese cannot be split into unaccusative and unergative verbs based on the syntactic distinctions.

A second possible explanation for the activation of the left posterior temporal gyrus is the reanalysis of the corresponding relationship between the passive theta role and the subject syntactic position for passive sentences. Den Ouden et al. [49], together with Makuuchi and Friederici [50], suggested that regarding theta roles on the same surface syntactic position, the lower the hierarchy position of a theta role, the more complicated the reanalysis procedure. The research mentioned here also pointed out that the left posterior temporal gyrus was related to the neural processing of the reanalysis. In the current study, though both are positioned at the same syntactic position, the subject of Mandarin passive sentences is assigned to a patient theta role, but that of Mandarin unergative sentences is assigned an agent theta role, and the patient at a higher theta role hierarchy position compared to the patient. Therefore, the neural mechanism of the reanalysis for the subject in the passive sentences is more complicated than that of the reanalysis for the subject in the unergative sentences, which then suggests that the activation of the left posterior temporal gyrus revealed in the current study seems to indicate that this region reflects the reanalysis of the corresponding relationship between the passive theta role and the subject syntactic position.

The third reason related to the left posterior temporal gyrus is the verb form or the abstract semantic processing. A study on spatiotemporal maps of past-tense verb inflection [37] found that at approximately 340 ms, irregular past-tense verb inflection evoked greater response modulation in left occipitotemporal cortex compared with regular ones, suggesting that left occipitotemporal cortex was associated with complex verb form processing. In the present study, passive verbs have irregular forms relative to those of the unaccusative and unergative ones as they consist of the preposition *被* and corresponding transitive verbs. Consequently, the activation of the LSTG in our research may reflect the irregular forms processing for passive verbs. In addition, research on left-hemisphere stroke patients [51] found that damage to the left anterior middle temporal gyrus significantly impaired the processing of abstract semantic information. The finding supported the view that abstract semantic processing relied on the left anterior middle temporal gyrus. Relative to deep unaccusative sentences and unergative sentences, the passive ones embrace the abstract semantics of suffering. Therefore, with reference to prior research, the LSTG found here may also be associated with the abstract semantic processing of passive sentences.

What should be further pointed out is that contrasted with Mandarin deep unaccusative sentences, Mandarin passive sentences also elicited more activation in the LSTG, while Mandarin deep unaccusative sentences induced no significant extra activation compared to Mandarin unergative sentences. These results show that the neural processing of the reanalysis for the subject in the deep unaccusative sentences is as complex as that of the reanalysis for the subject in the unergative sentences, but is different from that of the reanalysis for the subject in the passive sentences. Consequently, for Mandarin Chinese, the theta roles assigned to the unaccusative verbs and unergative verbs are at the same hierarchy position, which indicates that the arguments of Mandarin unaccusative verbs and unergative verbs are base-generated in the same syntactic position. Therefore, there is no syntactic difference between these two types of verbs.

In conclusion, the analyses of the LSTG above suggest that, for Mandarin Chinese, both deep unaccusative sentences and unergative sentences are base-generated structures, and the arguments of unaccusative verbs and unergative verbs are originally generated in the same syntactic position.

### 4.2. The LMFG

In the present research, the LMFG was also identified in the comparison between Mandarin passive sentences and deep unaccusative sentences and Mandarin passive sentences and unergative sentences, and this region has been linked to selecting from multiple options [52]. For instance, Chan et al. [53] detected activation in the LMFG when comparing the processing of Chinese semantically ambiguous words to unambiguous ones, which accorded with the findings of Ketteler, Kastrau, Vohn and Huber [54], and the researchers declared that the LMFG reflected the semantic selection of ambiguous words. Moreover, the LMFG is also considered to be associated with syntactic form selection. Numerous prior studies have documented LMFG activation while processing an ambiguous structure (realized by two syntactic forms) as compared to a single, unambiguous structure [55]. Meltzer-Asscher, Schuchard and den Ouden [52] detected activation in LMFG in response to the processing of alternating verbs with two different syntactic structures compared to that of simple verbs with only one syntactic realization, which is similar to the finding of Malyutina and den Ouden [56]. Meanwhile, the short passive sentences in Mandarin Chinese appearing in the present study all have a corresponding long syntactic structure where the passive mark *被* must be followed by the external argument (e.g., the short passive sentences *学生被打了*/*The students were hit* has a corresponding long structure *学生被小偷打了*/*The students were hit by the thief*). Thus, like the alternating verbs mentioned above, passive verbs in Mandarin Chinese also have two different syntactic forms [57]. However, the deep unaccusative sentences in the present research, whose syntactic structure is NP-V, have no corresponding surface unaccusative structures in which the syntactic structure is V-NP, due to the influence of the adverb *全*/all. Mandarin unergative verbs only have one syntactic form of NP-V. Therefore, the passive sentences in the present experiment have more syntactic realization options than deep unaccusative and unergative ones, and this suggests that the LMFG referred here may be involved in the syntactic form selection of passive sentences in Mandarin Chinese.

Moreover, the LMFG may be linked to complex semantic processing, especially for semantic integration [58]. For example, a meta-analysis of *f*MRI studies showed that the LMFG was an important region linked to Mandarin semantic integration [59]. Meanwhile, for Mandarin Chinese, the basic semantics of passive sentences are based on the semantic integration of the preposition *被* and corresponding transitive verbs, although such semantic integration operation does not occur for deep unaccusative and unergative sentences.

In addition, the LMFG is also involved in attention and control. A study using transcranial magnetic stimulation (TMS) and electroencephalography (EEG) found that the LMFG was associated with selective attention tasks [60], and Eayrs, & Lavie found that this region was related to cognitive control [61]. As mentioned above, the syntactic structure where Mandarin Chinese passive verb appears is optional as this type of verb can appear in both long and short passive sentences, and selecting the short passive structure in the present research may need more attention and control. Consequently, the LFMG found here may reflect the selective attention and cognitive control needed to process the passive verb.

## 5. Conclusions and Limitations

The present research aimed to investigate the neural mechanisms of processing intransitive verbs in Mandarin Chinese. In contrast to previous *f*MRI studies, the *f*MRI results revealed that in Mandarin Chinese, deep unaccusative sentences did not require more involvement of brain regions than unergative sentences did for processing. The results indicate that the neural mechanisms of the syntactic processing of deep unaccusative and unergative sentences in Mandarin Chinese were highly similar and suggest that contrasted with the unergative sentences, the deep unaccusative sentences in Mandarin Chinese did not experience an extra syntactic movement during the generation process. Meanwhile, compared to the Mandarin deep unaccusative and unergative sentences, the Mandarin passive sentences, a structure involved in an additional syntactic movement during the generation process, which was proved by a previous *f*MRI study [31], elicited more activation in the LSTG and the LMFG. The LSTG reflected the syntactic movement and the reanalysis of the subject for passive sentences, indicating that, unlike passive sentences, deep unaccusative and unergative sentences in Mandarin Chinese were base-generated structures and did not experience an extra syntactic movement. The LMFG reflected the syntactic form selection and semantic integration of passive sentences in Mandarin Chinese. In conclusion, the results of the present research suggest that the syntactic processing neural mechanism of Mandarin Chinese unaccusative verbs is highly similar to that of unergative verbs. The finding further indicates that intransitive verbs in Mandarin Chinese could not be divided into unaccusative and unergative categories, according to the syntactic level, and that the Unaccusative Hypothesis does not hold for all languages.

One clear limitation of this study relates to the semantic processing of unaccusative verbs and unergative verbs. Semantic processing is an integral component of language processing [62,63,64]. The present study is devoted to the syntactic processing of unaccusative and unergative verbs, but the two verb classes’ semantic features are different [5,65]. Therefore, the semantic processing of unaccusative and unergative verbs should be considered in future research, despite the control of the semantic factor in this present research attained by maintaining the consistency of the subject animacy of these two types of verbs. Another limitation was the experimental paradigm as the processing of verbs in a sentence can be modulated by context information. Future studies should address this limitation by designing a context-free paradigm using lexical judgment or picture–word matching tasks. Additionally, although many *f*MRI studies [21,22,42,53,58] compare the similarities or differences of processing mechanisms of different language structures by comparing the similarities or differences of the brain activation when processing them, some research claims that the *f*MRI technique has limitations [66], and some research points out that brain state may influence the *f*MRI results [67], which indicates that *f*MRI may not be a direct enough measure to explore brain activity of language processing. Therefore, faced with the debate on whether intransitive verbs in Mandarin Chinese can be split into unaccusative and unergative ones syntactically, other techniques such as ERP and eye-tracking should be adopted in follow-up studies.

## Figures and Tables

**Table 1:** Sample sentences of each condition in the present experiment.

Conditions	Semantic Types	Examples
Unaccusative sentence	Correct	客人 全 来了。 visitors all come Visitors all came.
	Incorrect	宇宙 全 来了。 the universe all come The universe all came.
Unergative sentence	Correct	孩子 全 哭了。 children all cry Children all cried.
	Incorrect	椅子 全 哭了。 chairs all cry Chairs all cried.
Passive sentence	Correct	学生 被 打了。 students bei hit Students was hit.
	Incorrect	月亮 被 打了。 the moon bei hit The moon was hit.
Filler sentence	Correct	枫叶 全 红了。 maple leaves all red Maple leaves all became red.
	Incorrect	学校 全 红了。 schools all red Schools all became red.

**Table 2:** Regions of different activation for unaccusative, unergative, and passive sentences compared to control task.

Conditions	Brain Regions	X	Y	Z	T	*P-*_FWE_	Voxels
Unaccusative > Control	Left occipital lobe (Visual Assoc18)	−23	−93	−7	15.37	0.000	269
	Left fusiform (Fusiform 37)	−42	−60	−21	8.19	0.000	
	Left inferior frontal gyrus (BA44)	−46	9	25	10.58	0.000	301
	Left middle frontal gyrus (BA8)	−53	16	35	9.79	0.000	
	Left middle temporal gyrus (BA39)	−57	−48	11	7.18	0.007	23
	Left medial frontal gyrus (BA32)	−8	8	53	9.84	0.000	
	Left inferior parietal lobe (BA7)	−27	−56	42	9.60	0.000	54
	Left cerebellum posterior lobe	−5	−71	28	7.06	0.000	121
	Right lingual gyrus (Visual Assoc18)	26	−90	−7	10.02	0.001	139
	Right fusiform (Fusiform 37)	44	−60	−21	6.65	0.000	
	Right occipital lobe (BA19)	41	−67	−1	6.37	0.000	
	Right inferior frontal gyrus (BA9)	52	16	32	8.54	0.000	48
	Right cerebellum posterior lobe	7	−75	−28	7.30	0.000	121
Unergative > Control	Left occipital lobe (Visual Assoc18)	−23	−90	−7	10.96	0.000	301
	Left inferior temporal gyrus	−46	−60	−11	9.69	0.000	
	Left precentral gyrus (BA6)	−46	4	32	9.64	0.000	299
	Left inferior frontal gyrus (BA44)	−46	23	25	8.56	0.000	
	Left middle frontal gyrus	−35	1	42	8.25	0.000	
	Left medial frontal gyrus (BA6)	−8	4	56	9.10	0.000	81
	Left inferior parietal lobe (BA7)	−27	−56	42	8.94	0.001	37
	Left middle temporal gyrus (BA22)	−53	−48	11	7.40	0.002	31
	Left cerebellum posterior lobe	−8	−75	−28	11.08	0.000	84
	Right occipital lobe (Visual Assoc18)	29	−90	−7	10.46	0.000	146
	Right inferior frontal gyrus (BA44)	52	16	32	6.91	0.001	43
	Right cerebellum posterior lobe	7	−75	−28	9.18	0.000	84
Passive > Control	Left cerebellum posterior lobe	−5	−56	−18	12.85	0.000	632
	Left occipital lobe (Visual Assoc18)	−27	−90	−11	11.89	0.000	
	Left precentral gyrus (BA9/6)	−53	12	35	11.04	0.000	306
	Left inferior frontal gyrus (BA44)	−42	19	18	9.25	0.000	
	Left inferior parietal lobe (BA7)	−27	−56	42	10.92	0.001	42
	Left medial frontal gyrus (BA6)	−5	8	53	9.06	0.000	139
	Right occipital lobe (Visual Assoc18)	29	−90	−7	9.65	0.000	90
	Right inferior frontal gyrus (BA44)	52	16	35	6.72	0.009	22
	Right cerebellum posterior lobe	33	−48	−28	6.51	0.000	68

Table 2 shows regions of different activation for unaccusative, unergative, and passive sentences compared to control task in Montreal Neurological Institute (MNI) coordinates (cluster FWE correction, *p* < 0.05). T-values, cluster size (Voxels), and cluster-level *p*-values are reported. Notes: Unaccusative: deep unaccusative sentences; Unergative: unergative sentences; Passive: passive sentences; Control: control task.

**Table 3:** Regions of activation for the null conjunction of the comparison between passive sentences and deep unaccusative sentences and the comparison between passive sentences and unergative sentences.

Condition	Brain Regions	X	Y	Z	T	*P*_-FWE_	Voxels
Conjunction	Left middle frontal gyrus (BA9)	−31	34	42	5.34	0.025	74
	Left middle frontal gyrus	−23	8	60	4.66	0.025	
	Left superior temporal gyrus (BA21/22)	−50	−33	7	4.82	0.027	70

Table 3 shows regions of activation for the null conjunction of the comparison between passive sentences and deep unaccusative sentences and the comparison between passive sentences and unergative sentences in Montreal Neurological Institute (MNI) coordinates (cluster FWE correction, *p* < 0.05). T-values, cluster size (Voxels), and cluster-level *p*-values are reported. Notes: Conjunction: (passive sentences > deep unaccusative sentences) ∩ (passive sentences > unergative sentences).

## Data Availability

The data presented in this study are available on request from the first author and the corresponding author.
